# Lipidomic Biomarkers in Polycystic Ovary Syndrome and Endometrial Cancer

**DOI:** 10.3390/ijms21134753

**Published:** 2020-07-03

**Authors:** Mohamad Nasir Shafiee, Catharine A. Ortori, David A. Barrett, Nigel P. Mongan, Jafaru Abu, William Atiomo

**Affiliations:** 1 Department of Obstetrics and Gynaecology, Faculty of Medicine, Universiti Kebangsaan Malaysia, Cheras, Kuala Lumpur 56000, Malaysia; nasirshafiee@ukm.edu.my; 2 Division of Advanced Materials and Healthcare Technologies School of Pharmacy, Centre for Analytical Bioscience, University of Nottingham, University Park, Nottingham NG7 2RD, UK; catherine.ortori@nottingham.ac.uk (C.A.O.); david.barrett@nottingham.ac.uk (D.A.B.); 3 University of Nottingham Biodiscovery Institute, University of Nottingham LE12 5RD, UK; svznpm@exmail.nottingham.ac.uk; 4 School of Veterinary Medicine and Science, University of Nottingham LE12 5RD, UK; 5 Department of Obstetrics and Gynaecology, City Hospital, Nottingham University Hospital, Nottingham NG5 1PB, UK; jafaru.abu@nuh.nhs.uk; 6 Division of Obstetrics and Gynaecology and Child Health, School of Medicine, Faculty of Medicine and Health Sciences, University of Nottingham, Queen’s Medical Centre, Nottingham University Hospital, Derby Road, Nottingham NG7 2UH, UK

**Keywords:** endometrial cancer, lipidomic, biomarkers, PCOS

## Abstract

Women with polycystic ovary syndrome (PCOS) are more likely to develop endometrial cancer (EC). The molecular mechanisms which increase the risk of EC in PCOS are unclear. Derangements in lipid metabolism are associated with EC, but there have been no studies, investigating if this might increase the risk of EC in PCOS. This was a cross-sectional study of 102 women in three groups of 34 (PCOS, EC and controls) at Nottingham University Hospital, UK. All participants had clinical assessments, followed by obtaining plasma and endometrial tissue samples. Lipidomic analyses were performed using liquid chromatography (LC) coupled with high resolution mass spectrometry (HRMS) and the obtained lipid datasets were screened using standard software and databases. Using multivariate data analysis, there were no common markers found for EC and PCOS. However, on univariate analyses, both PCOS and EC endometrial tissue samples showed a significant decrease in monoacylglycerol 24:0 and capric acid compared to controls. Further studies are required to validate these findings and investigate the potential role of monoacylglycerol 24:0 and capric acid in the link between PCOS with EC.

## 1. Introduction

Endometrial cancer (EC) is a common cause of death from female cancers globally [1]. The incidence of Type 1 EC, which is more preponderant amongst women of late reproductive age, is rising. Since the 1990s, the incidence of EC has doubled [1]. It has also been predicted that the number of EC cases will increase by 50–100% by 2030 because of rising obesity rates [2]. Research to identify and prevent EC in women at increased risk is therefore vital. Although increasing age itself is one of the important risk factors for EC, endocrine and metabolic factors also play an integral part in EC development. Chronic unopposed oestrogen exposure to the endometrium, which is similarly present in women with polycystic ovary syndrome (PCOS) and EC, is thought to be a key mechanism which increases the risk of PCOS women developing EC [3], especially as women with PCOS are 3–4 times more likely to develop EC compared to women without PCOS [4,5].

Apart from chronic unopposed oestrogen exposure to the endometrium in women with PCOS, other potential molecular mechanisms which are thought to increase the risk of EC in women with PCOS include (i) insulin resistance and (ii) endometrial overexpression of insulin like growth factor-1 (*IGF1*), IGF binding protein-1 (*IGFBP1*), *PTEN* genes [6], sterol regulatory binding protein-1 (*SREBP1*) and adiponectin [7]. Nair et al. [7], reported that derangements in adipocyte, lipid and fatty acid metabolism are associated with increased EC risk either through release of fatty acids from cancer-associated adipocytes which are used for intracellular energy production in cancer cells or through the promotion of inflammation. Prior to the commencement of this study, there had however been no reported studies investigating how endometrial lipid profiles in women with PCOS compare to those in women with EC.

Metabolomics is an increasingly important tool for oncology research, especially as it aims to improve the early detection of pre-invasive lesions, triage neoplastic differentiation, monitor response to cancer treatment, facilitate pharmacodynamic analysis and determine prognosis of disease. It has been shown that in general, tumour growth and activity correlate to increased phospholipid, and glycolytic capacity, with elevated choline containing compounds, phosphocholine, high glutaminolytic activity and overexpression of the glycolytic isoenzymes, pyruvate kinase M2 [8]. Bathen et al., 2000 [9] reported an accuracy of 83% in differentiating between cancer and normal samples by analysing lipid metabolic profiles using nuclear magnetic resonance. However, it is also acknowledged that different tumours may have distinct metabolites properties, and this may be difficult to determine [10]. An epidemiological case-control study identified 15 amino acids, 45 acylcarnitines and 9 fatty acid metabolites differentially expressed in endometrial cancer in comparison to the normal population. Among the control group, obesity itself produced a significant change in metabolite profiles with elevated valine, octenoylcarnitine, palmitic acid, oleic acid and stearic acid [11]. Even after adjusting for obesity, the study reported a significant difference in spectrometric profiles of metabolites, namely C5-acylcarnitine, octenoylcarnitine, decatrienoylcarnitine and linoleic acid, which were found to be lower in the endometrial cancer group. As women with PCOS have a three to four-fold increased risk of EC, investigating endometrial lipid profiles of women with PCOS and EC may, therefore, help us better understand the mechanisms which increase the risk of EC in PCOS.

The aim of this study, therefore, was to investigate whether lipid profiles were similarly altered in the endometrium of women with EC (without PCOS) and women with PCOS (without EC), compared to a control group of women who did not have a diagnosis of EC nor PCOS, by measuring the endometrial lipidomic profiles in women with PCOS, EC and controls. This would be an important finding as it will offer the potential to facilitate screening and early diagnosis of pre-cancerous cells in the endometrium, thus preventing the development of EC in women with PCOS. Although a range of factors have been identified previously, which were thought to increase the risk of EC in PCOS, there had been no previously published studies investigating endometrial lipid profiles in women with PCOS and EC, despite the known changes in serum lipid profiles in PCOS and recent associations between changes in lipid metabolism and increased EC risk. Our null hypothesis was that there would be no difference in endometrial lipid profiles between groups.

## 2. Results

### 2.1. Demography

The demographic data are shown in Table 1. There was no statistically significant difference in the mean age between PCOS and controls. However, the mean age was significantly higher in women with EC (63.44 ± 10.07 years) compared to PCOS (31.8 ± 5.97 years) and controls (43.68 ± 13.12 years) [6]. PCOS women and controls had a similar body mass index (BMI) and waist–hip ratio (WHR), but these were higher in women with EC. Although at recruitment, only PCOS women in their proliferative stage of the menstrual cycle (based on the date of their last menstrual period) were included, on histological examination of the endometrial biopsies, of the 34 samples, three women were in the secretory phase and eight samples were inadequate for histological classification. In the EC group, participants who were diagnosed as endometroid adenocarcinoma subtype and scheduled for surgical staging by either laparoscopy or laparotomy were recruited. Moderately differentiated endometroid adenocarcinoma was more prevalent in the EC group, 44.1%, followed by poorly differentiated and well differentiated, 29.4% and 26.5%, respectively. Biochemically, only fasting glucose was significantly different among the groups (higher in EC women) with no statistically significant difference noted in the other parameters measured (*p* > 0.05).

### 2.2. Lipidomic Profiling

**Figure 1:**
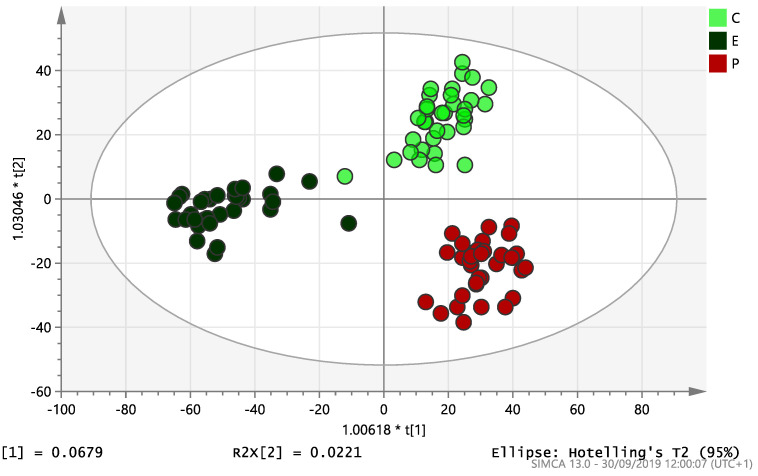
Multivariate analysis based on all detected ions from endometrial tissue samples: OPLS-DA score plot of control (C, *n* = 34), endometrial cancer (E, *n* = 34) and PCOS (P, *n* = 34). R2X(cum) = 0.286 and Q2(cum) = 0.514.

**Figure 2:**
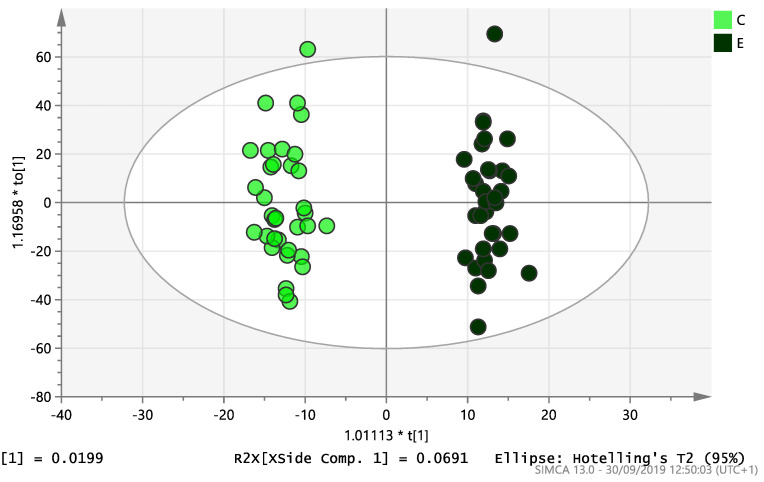
Multivariate analysis based on all detected ions from plasma samples: OPLS-DA score plot of control (C, *n* = 34) and endometrial cancer (E, *n* = 34), R2X(cum) = 0.298 and Q2(cum) = 0.296.

**Figure 3:**
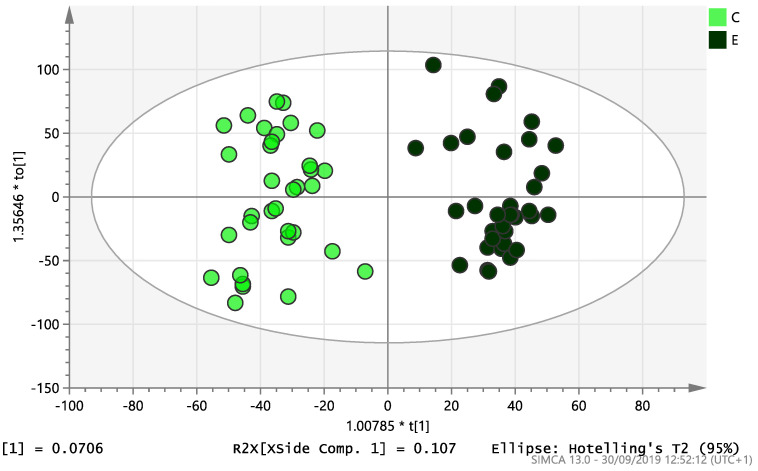
Multivariate analysis based on all detected ions from endometrial tissue samples: OPLS-DA score plot of control (C, *n* = 34) and endometrial cancer (E, *n* = 34). R2X(cum) = 0.195 and Q2(cum) = 0.583.

**Figure 4:**
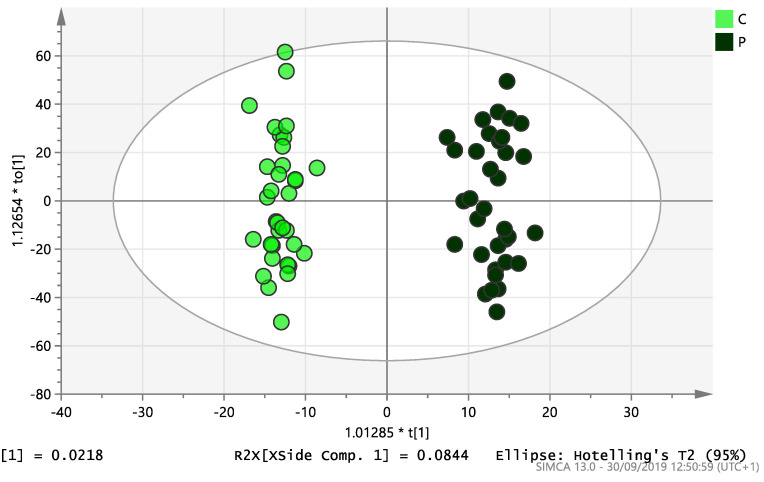
Multivariate analysis based on all detected ions from plasma samples: OPLS-DA score plot of control (C, *n* = 34) and PCOS (P, *n* = 34). R2X(cum) = 0.196 and Q2(cum) = 0.312.

**Figure 5:**
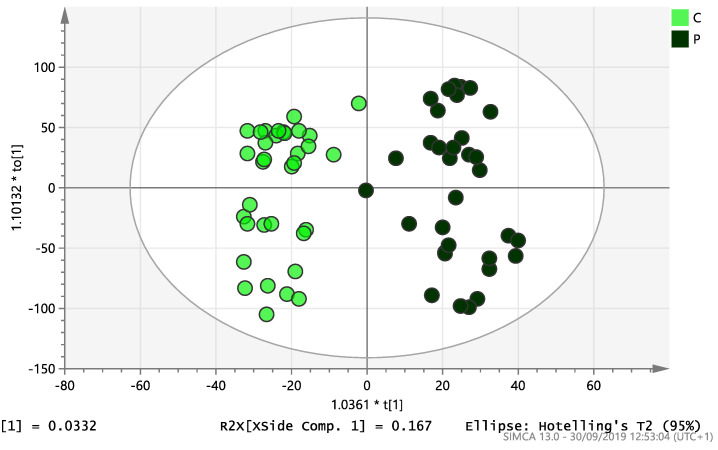
Multivariate analysis based on all detected ions from endometrial tissue. samples: OPLS-DA score plot of control (C, *n* = 34) and PCOS (P, *n* = 34). R2X(cum) = 0.228 and Q2(cum) = 0.433.

On initial analysis, by LC-MS, the lipidomics data from the samples identified >20,000 peaks of lipid compounds. We then used multivariate analysis (MVA) and univariate analysis (UVA) to define lipids which were significantly different between the three study groups (control, EC and PCOS). Following MVA, we did not identify any visible separation between the three study groups in a principal component analysis (PCA). Further orthogonal projections to latent structures discriminant analysis (OPLS-DA) model (Figure 1), however, showed a complete separation of detected ions in tissue from control, PCOS and EC groups, with a R2X(cum)-value of 0.286 and a Q2(cum)-value of 0.514, which are typical for clinical samples. We also found that the CV-ANOVA *p*-value was highly significant (*p* = 6 × 10^−7^), which indicates a good model. Separations were also identified in detected ions in plasma and tissue from control vs. EC subjects (Figure 2 and Figure 3) and control and PCOS subjects (Figure 4 and Figure 5).

Table 2 shows the significantly changed lipid compounds that were decreased or increased between PCOS and control plasma samples on univariate analysis. Heptadecanoic acid (17:1), palmitoleic acid (16:1), pentadecnoic acid (15:1), palmitic acid (16:0), Heptadecanoic acid (17:0), Myristic acid (14:0), Docosatrienoic acid (22:3) and Eicosadienoic acid (20:2) were decreased and a steroid (dihydrotestosterone or isomer) sulphate, hexacosahexaenoic acid FA (26:6) and a steroid (testosterone or isomer) sulphate were increased in PCOS compared with control plasma. We however, did not identify any lipid compounds that were obviously, similarly increased or decreased in both PCOS and EC women compared with controls. Dihydrotestosterone and testosterone were raised as expected in PCOS compared to EC and controls.

**Figure 6:**
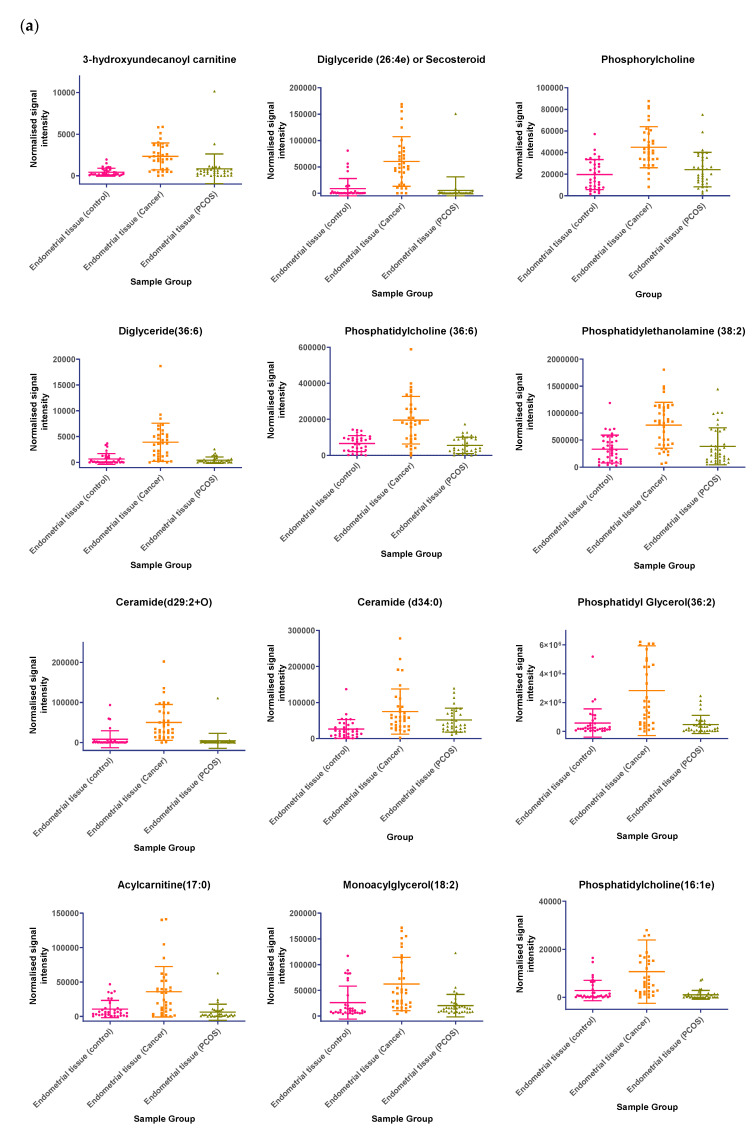

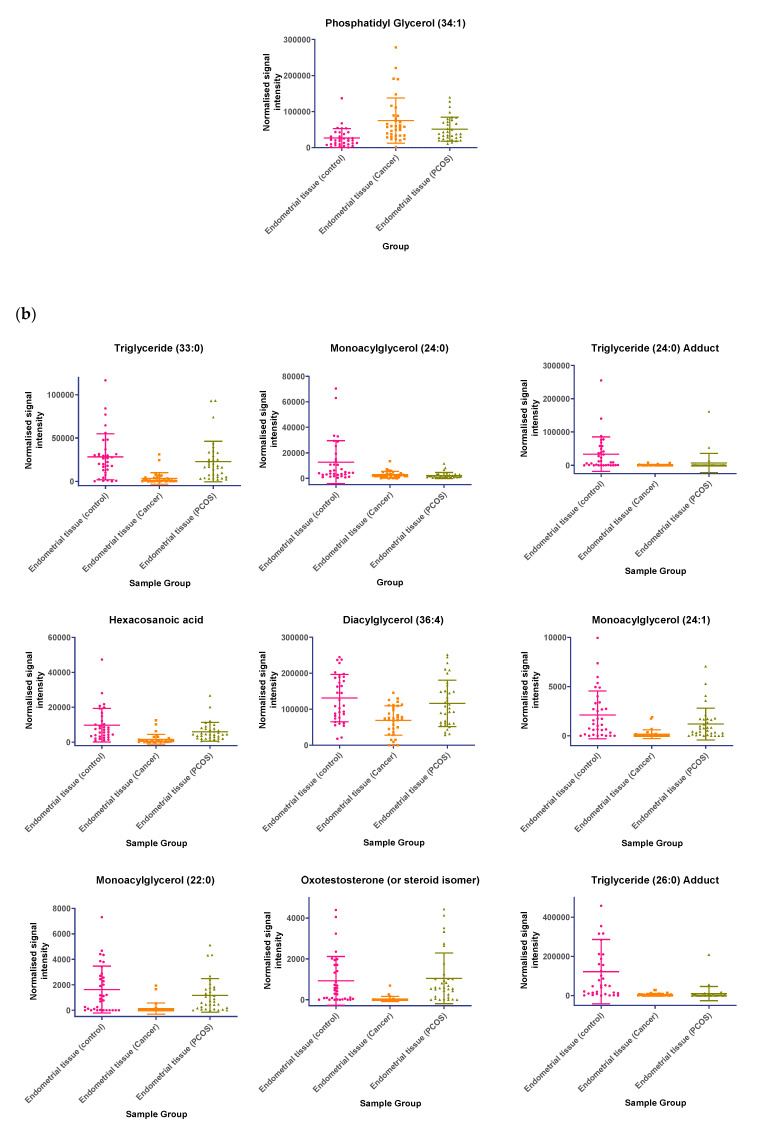

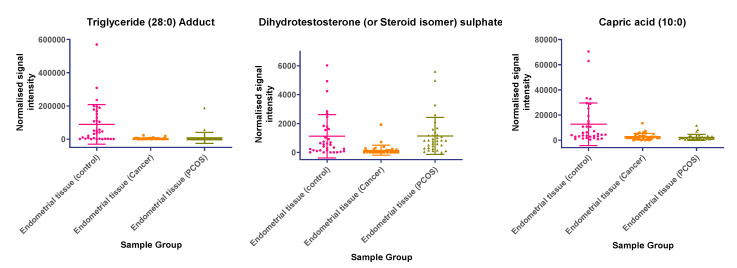
(**a**) Compounds that significantly increase in concentration in the endometrial cancer group; (**b**) Compounds that significantly decrease in concentration in the endometrial cancer group. Scatter dot plots of example potential biomarkers in endometrial tissue samples. All shown biomarkers fulfil criteria of both multivariate analysis (MVA) and univariate analysis (UVA). MVA criteria were a Variable Importance Projection (VIP) value > 1 in the OPLS-DA model shown in Figure 1. Criteria for UVA were a *q*-value < 0.05 (control vs. endometrial cancer only), for all control vs. EC, Q > 0.05 and a fold change <0.8 or >1.2. Dots show the normalised intensity metabolites from control endometrial cancer and PCOS tissue samples (*n* = 34), samples.

In tissue samples (Table 3), the following lipid compounds were increased in EC compared with control tissue; hydroxyundecanoyl carnitine, phosphorylcholine, diglyceride (26:4e) or secosteroid, phosphatidylcholine (36:6), phosphatidylethanolamine (38:2), ceramide (d29:2+O), phosphatidylethanolamine (36:6e), diglyceride (36:6), ceramide (d34:0), phosphatidyl glycerol (36:2), acylcarnitine (17:0), monoacylglycerol (18:2), phosphatidylcholine (16:1e). On the other hand, the following were decreased; triglyceride (33:0), monoacylglycerol (24:0), hexacosanoic acid, diacylglycerol (36:4), monoacylglycerol (24:1), monoacylglycerol (22:0), sterol at C_27_H_48_O_5_, monoacylglycerol (22:4), oxotestosterone (or steroid isomer), triglyceride (28:0) adduct, monoacylglycerol (22:2), monoacylglycerol (24:4), dihydrotestosterone (or steroid isomer) sulphate, triglyceride (24:0), capric acid. A scatter dot plot of these biomarkers, from tissue samples (Figure 6) did not identify any lipid compounds that were clearly similarly increased or decreased in PCOS and EC compared with controls apart from decreased monoacyl glycerol 24:0 and capric acid 10:0.

## 3. Discussion

Our results showed that although there were two metabolites (monoacylglycerol 24:0 and capric acid) similarly decreased in PCOS and EC tissue compared to controls on univariate analysis, no common markers were found for the EC and the PCOS samples using OPLS-DA models. Lipid compounds were also identified, which were significantly decreased or increased between EC and control tissue samples; there was, however, no obvious correlation with plasma results and therefore, they are not currently potential biomarkers for EC in PCOS without further research. However, although women with EC had increased age, BMI and fasting glucose levels, baseline metabolic characteristics (BMI, markers of insulin resistance and lipid profiles) were not statistically different between PCOS and control women. The potential significance of this is the fact that women with PCOS were showing comparable tissue changes in these two metabolites (monoacylglycerol 24:0 and capric acid), to women with EC, at an earlier age and lower BMI. If validated in future studies and correlated with plasma results, they, therefore, could act as potential early biomarkers for future development of EC in women with PCOS.

As the baseline sample size calculation for this study was based on the ability to detect an outcome from a previously published study on *IGF1* gene expression [6], we are unable to exclude the possibility of a type 1 statistical error in the observed differences in monoacylglycerol 24:0 and capric acid in PCOS and EC tissue compared to controls. We were, therefore, unable to confidently reject our null hypothesis that there would be no endometrial lipid profile, common to EC and PCOS endometrium but different from that found in the endometrium of control women.

It is also difficult at this stage to speculate on how the decreased monoacylglycerol 24:0 and capric acid found in endometrial tissue from women with EC and PCOS compared with controls may explain the link between PCOS and EC and further research is required. We were, for example, unable to identify any previously published studies measuring caproic acid levels in women with PCOS or EC. Caproic acid (Hexanoic acid) is, however, the carboxylic acid derived from hexane and is thought to be involved in cell signalling, fuel and energy storage, fuel or energy source, membrane integrity/stability, vasorelaxation and has inflammatory effects [13,14,15]. Interestingly, there is evidence that caproic acid can inhibit proliferation of a number of different cancer cell types in vitro [16], suggesting that a decrease in physiological levels of caproic acid may have a pro-oncogenic effect. A decrease in physiological levels of caproic acid may have a pro-inflammatory effect which is relevant in cancer. Notably, in plants, caproic acid has been shown to influence expression of redox-sensitive genes and reduce oxidative stress [17]. NQO1 is another key regulator of cellular redox status and was recently shown to be increased in the endometrium of women with EC or PCOS [18]. There is also increasing evidence supporting a role for NQO1 in metabolic regulation [19,20], including lipid metabolism. *NQO1* expression is elevated in adipose tissue, is reduced by diet-induced weight loss and may contribute to the physiological consequences of obesity [21]. Consistent with this, *NQO1*^−/−^ mice accumulate significantly less abdominal adipose tissue, with concomitant increase in liver triglycerides and reduction in liver glycogen [20]. The potential functional interaction of increased *NQO1* expression and decreased caproic acid in EC should therefore be examined.

Although we were also unable to identify any previous studies on monoacylglycerol and EC tissue, monoacylglycerol is a bioactive compound and its biosynthesis and metabolism modulate a range of cellular processes including proliferation, migration, and apoptosis. Monoacylglycerol lipase levels have been shown to be overexpressed in EC [22], which is consistent with the lower levels of monoacylglycerol identified in our study. On the other hand, higher levels of serum monoacylglycerol 1-oleoylglycerol [23] were found in recurrent cases of endometrial cancer compared with non-recurrent cases, but how this finding fits in our results is however currently unclear.

As with the case of EC tissue, we could not find any previous studies on whether there is any link between decreased levels of monoacylglycerol and endometrial tissues obtained from women with PCOS. We did, however, find one study [24] which showed that plasma monoacylglycerol levels were up to 5-fold higher in women with PCOS. As the samples were from plasma and the direction of change differed from our results (plasma in contrast to tissue; higher instead of lower), it is again challenging to speculate on any consistent patterns.

The molecular mechanisms which lead to an increased risk of EC in PCOS probably arise as a result of complex interactions between a range of systems and pathways, including chronic unopposed oestrogen, the insulin and the lipid pathways and tumour regulatory genes. More recent research has also suggested a potential role for micro-RNAs [25], excess androgens and Visfatin [26]. More studies are, therefore, required to validate our findings and to investigate how these various pathways interact in such a way as to increase EC risk in women with PCOS.

The strengths of our study were the detailed clinical description of study participants, the originality of the lipidomic approach and the rigorous methodological approach involved. The limitations, however, include the histological heterogeneity of tissue samples obtained from both EC and PCOS women and the age and BMI differences between the groups. In the context of the pilot nature of this study, these confounders should be addressed in any future validation studies. The age and BMI, however, were not statistically different between PCOS and control women. Obesity and type 2 diabetes are risk factors for EC which would explain the higher BMI and glucose in the EC group.

Although the baseline sample size calculations were based on the outcomes from another study on *IGF-1*, the overall sample size of 34 in each group was not unreasonable if considered as a pilot on which to base sample size calculations for future studies aiming to validate our findings. It would also have been ideal to have included the specificity and sensitivity of the tested model. However, this study was intended as a pilot to investigate possible metabolic perturbations which might be common between PCOS and EC. We are, therefore, not proposing that the study data be used as a predictive model for diagnostic purposes, and hence, we believe that the calculations of specificity and sensitivity were not relevant. In any case, there were insufficient subjects to allow for the building of “training” and “test” sets to validate a prediction model from the data, so calculations of specificity and sensitivity would not have any real meaning for this pilot dataset. It could also be argued that to improve the understanding about which groups of metabolites and pathways were involved in PCOS and EC, we could have performed a metabolite group enrichment analysis and/or a clustering exploratory analysis. However, our study was primarily done to look for any common metabolic changes in both PCOS and EC. Tools to enable mapping of the complex lipid pathways, based on individual lipid entities, are not yet fully established. Hence, we could not perform detailed pathway analysis, but we have outlined the changes in levels of lipid families, such as fatty acids, glycerides and phospholipids, in our results.

## 4. Materials and Methods

### 4.1. Study Design

This was a two-year cross-sectional study conducted at Nottingham University Hospital, NHS Trust, in the United Kingdom. Recruitment was conducted in the Department of Obstetrics and Gynaecology and Gynaecologic Oncology Unit from July 2013 to February 2014, following research ethics approval (13/EM/0119, 8 April 2013). Parts of this research project on insulin signalling pathways [6] and SREBP expression [27] have been previously published.

### 4.2. Recruitment

Patient recruitment has been previously described [6,27]. Briefly, 102 women in total were recruited into three groups of 34 each (PCOS, EC and controls). PCOS was diagnosed based on the Rotterdam criteria [28]. Their ages ranged between 18 to 45 years. The women were not on any form of hormonal and infertility therapy and were not pregnant. In the women with EC, participants diagnosed with the endometroid adenocarcinoma subtype and scheduled for surgical staging by either laparoscopy or laparotomy were recruited. For the control group, women with benign gynaecology problems undergoing surgery (i.e., to remove uterine fibroids, hysterectomy, tubal ligation and remove benign ovarian cysts) were recruited. All participants were identified in the Gynaecology clinic and Gynaecology Oncology clinic at the Nottingham University Hospitals, NHS Trust, United Kingdom.

After obtaining informed consent, the participants were assessed clinically and biochemically. Baseline demographic details (i.e., ethnicity, age, medical, reproductive and family history) and clinical physical assessments (i.e., blood pressure, BMI, Ferriman–Gallwey scoring, measurements of the hip and waist circumference) were performed. Serum blood samples for biochemical variables were then obtained. Following that, an outpatient endometrial tissue sampling was taken from the women in the PCOS subgroup with a Pipelle^®^ endometrial catheter. In the EC and controls groups, the endometrial tissue samples were obtained in the operating theatres either at the time of hysterectomy or hysteroscopy. The samples obtained were then frozen at −80 °C in liquid nitrogen and stored for future analysis.

### 4.3. Endocrine and Metabolic Assays

The biochemical assays were processed in the clinical chemical department at Nottingham University Hospital, NHS Trust. Cobas 8000^®^ System (Roche, UK) was used to measure the levels of fasting blood sugar, high density lipoprotein (HDL), low density lipoprotein (LDL) and triglycerides. The Architect 12000SR^®^ equipment (Abbott, UK) was used for immunoassays of luteinizing and follicular stimulating hormone (LH and FSH), oestradiol and testosterone levels. Serial verification methods, namely precision, accuracy, linearity and method comparison studies were performed for validation and they had intra-assay CVs of 2.3–4.1%, 2.2–2.6%; 4.4–10%, and 2.9–5.0%, respectively; and inter-assay CVs of 5.3–6.6% and 4.9–6.3%, 8.8–12.4%, and 9.1–10.9%, respectively [6,27].

Untargeted lipidomic analysis was performed using liquid chromatography and accurate mass high resolution mass spectrometry, based on a previously reported method [29]. For plasma and endometrial tissue lipidomics analysis, the samples used for quality control (QC) were created by pooling equal volumes from all samples and injected 6 times at the start and end of the sequence and every ten samples. The samples were all randomized before extraction and injection. Pooled QC samples were then used to evaluate the analytical performance. The raw data obtained were acquired using Xcalibur software (Thermo Scientific, Hemel Hempstead, UK). Pre-processing of the data was performed with the Progenesis QI software (Nonlinear Dynamics, Newcastle upon Tyne, UK). The process of peak picking was undertaken using auto threshold and an automatically chosen QC injection was used as reference for chromatographic alignment and normalisation. The peak intensities were normalised to all compounds. This involved calculating abundance ratios between each sample and the reference run for all compounds. The validity of the lipidomics analysis was confirmed by monitoring in the study QC samples (*n* = 21) a representative selection of specific lipids. The RSD% of lipid peak areas (5–17.6%) and retention times (0.23–2.37) were within generally accepted guidelines for metabolomics analysis (Supplementary Table S1). Biomarkers that were filtered by univariate or multivariate statistical analysis were identified by searching for their *m/z* values in the following databases (Human Metabolome Database (www.hmdb.ca), METLIN Metabolomics Database (www.metlin.scripps.edu) and Lipid Maps (www.lipidmaps.org)). Putative identifications were based on mass error < 5 ppm, relative retention time within a lipid family and demonstrated or expected presence in the human organism. The confidence of metabolite identification was categorized using identification classes [12]. Univariate analysis (UVA) was performed using Student’s *t*-test. False discovery was determined using the two-stage linear step-up procedure of Benjamini, Krieger and Yekutieli [30], with Q at <5% using GraphPad Prism v 8. For further advanced multivariate analysis (MVA), the datasets were imported to SIMCA (Version 13.0, Umetrics AB, Umea, Sweden) for principal component analysis and subsequent partial least squares discriminant analysis.

Apart from the statistical methods used for the lipidomic analysis outlined above, the overall sample size considerations and statistical methods used for this cohort have been previously published [6] and calculated to detect a 40% difference in *IGF1* gene expression in women with PCOS compared with controls and women with PCOS compared with endometrial cancer; this was the primary outcome measure of the study on which the samples used for the current study were derived.

## 5. Conclusions

In conclusion, no strongly predictive model common to both PCOS and EC was identified by lipidomic analysis of endometrial biopsies and plasma taken from 34 women with EC, 34 PCOS women and 34 control women. However, on univariate analyses, both PCOS and EC endometrial tissue samples showed a significant decrease in monoacylglycerol 24:0 and capric acid levels as compared to controls. Further studies are, however, required to validate these findings and explore how the other lipid changes found in this study may explain the link between PCOS and EC. Given the complexity of the pathogenesis of PCOS, simultaneous investigation of how these molecules link with known genetic pathways and environmental factors which increase the risk of EC is required to reduce the mortality from EC in PCOS women.

## Figures and Tables

**Table 1:** Patient Demographics.

Variable (Mean ± SD)	Polycystic Ovary Syndrome (*N* = 34)	Endometrial Cancer (*N* = 34)	Control (*N* = 34)
Age (Years)	31.8 ± 5.97	63.44 ± 10.07 *	43.68 ± 13.12
Menstrual phase	Proliferative = 34	Premenopause = 4 Perimenopause = 13 Postmenopause = 17	Premenopause = 20 Perimenopause = 14
BMI (kg/m^2^)	29.28 ± 2.91	32.22± 5.70 *	28.58 ± 2.62
WHR	0.88 ± 0.03	0.91 ± 0.04 *	0.85 ± 0.02
Systolic BP	133.4 ± 7.09	146.7 ± 10.7 *	134.5 ± 8.4
Diastolic BP	82.2 ± 7.95	87.5 ± 6.9	80.56 ± 7.0
Fasting Glucose (mmol/L)	5.16 ± 0.78	6.3 ± 1.5 *	4.9 ± 0.5
HOMA-IR	0.25 ± 0.39	0.28 ± 0.33	0.17 ± 0.10
LDL (mmol/L)	2.74 ± 0.77	2.57 ± 1.07	2.75 ± 0.80
HDL (mmol/L)	1.44 ± 0.33	1.63 ± 0.34	1.47 ± 0.33
TG (mmol/L)	1.43 ± 0.51	1.51 ± 0.54	1.38 ± 0.60
Total Cholesterol	4.73 ± 0.91	4.59 ± 1.39	4.94 ± 0.98

*—*p*-value < 0.001; HOMA-IR—homeostatic model assessment of insulin resistance; BP—blood pressure; SD—standard deviation; BMI—body mass index; WHR—waist–hip ratio; TG—triglycerides; LDL—low density lipoproteins; HDL—high density lipoproteins.

**Table 2:** Significantly changed lipid compounds that decreased or increased between PCOS and control (plasma samples). Discovery determined using the two-stage linear step-up procedure of Benjamini, Krieger and Yekutieli, with Q = 5%. Each row was analysed individually, without assuming a consistent SD. * Metabolite identification confidence score as described elsewhere [12].

Name	Identity Confidence Score *	*m/z*	Retention Time (min)	Increase (↑) or Decrease (↓)	Fold Change	*t*-Test (*p*)	Q-Value
Heptadecanoic acid (17:1)	2	267.232	1.70	↓	0.59	5.27 × 10^−6^	7.84 × 10^−3^
Palmitoleic acid (16:1)	2	253.217	1.52	↓	0.57	5.60 × 10^−6^	7.84 × 10^−3^
Pentadecenoic acid (15:1)	2	239.201	1.35	↓	0.65	4.76 × 10^−5^	2.16 × 10^−2^
Palmitic acid (16:0)	2	255.233	1.81	↓	0.74	6.30 × 10^−5^	7.84 × 10^−3^
Heptadecanoic acid (17:0)	2	269.248	2.03	↓	0.78	1.07 × 10^−4^	3.21 × 10^−2^
Myristic acid (14:0)	2	227.201	1.40	↓	0.65	1.41 × 10^−4^	3.95 × 10^−2^
Docosatrienoic acid (22:3)	2	333.279	2.11	↓	0.64	1.71 × 10^−4^	4.45 × 10^−2^
Eicosadienoic acid (20:2)	2	307.264	1.99	↓	0.70	3.79 × 10^−3^	2.16 × 10^−2^
Steroid (dihydrotestosterone or isomer) sulphate	4	369.172	0.45	↑	2.79	1.00 ×10^−5^	9.64 × 10^−3^
Hexacosahexaenoic acid (26:6)	2	383.297	2.05	↑	1.23	5.13 × 10^−5^	2.23 × 10^−2^
Steroid (testosterone or isomer) sulphate	4	367.158	0.43	↑	2.23	5.20 × 10^−5^	2.23 × 10^−2^

**Table 3:** Significantly changed lipid compounds that decreased or increased between endometrial cancer and control (tissue samples). * Metabolite identification confidence score as described elsewhere [12].

Name	Identity Confidence Score *	*m/z*	Retention Time (min)	Increase (↑) or Decrease (↓)	Fold Change	*t*-Test (*p*)	Q-Value
Hydroxyundecanoyl carnitine	4	363.290	0.64	↑	3.48	8.68 × 10^−9^	1.39 × 10^−4^
Phosphorylcholine	3	184.074	0.66	↑	2.29	3.38 × 10^−8^	1.71 × 10^−4^
Diglyceride (26:4e) or Secosteroid	4	480.405	3.56	↑	6.94	1.30 × 10^−7^	3.76 × 10^−4^
Phosphatidylcholine (36:6)	3	778.539	4.82	↑	2.99	7.26 × 10^−7^	6.26 × 10^−4^
Phosphatidylethanolamine (38:2)	3	772.586	6.31	↑	2.45	2.00 × 10^−6^	9.66 × 10^−4^
Ceramide (d29:2+O)	3	482.421	4.47	↑	6.29	5.00 × 10^−6^	1.53 × 10^−3^
Phosphatidylethanolamine (36:6e)	3	722.511	4.43	↑	2.46	5.00 × 10^−6^	1.53 × 10^−3^
Diglyceride (36:6)	3	630.511	4.15	↑	5.71	7.00 × 10^−6^	1.68 × 10^−3^
Ceramide (d34:0)	3	540.534	4.59	↑	2.82	9.60 × 10^−5^	7.39 × 10^−3^
Phosphatidyl Glycerol (36:2)	3	773.533	2.84	↑	4.84	1.57 × 10^−4^	1.01 × 10^−2^
Acylcarnitine (17:0)	3	414.358	2.72	↑	3.39	3.21 × 10^−4^	1.45 × 10^−2^
Monoacylglycerol (18:2)	3	372.311	1.69	↑	2.38	9.79 × 10^−4^	2.69 × 10^−2^
Phosphatidylcholine (16:1e)	3	538.352	1.99	↑	3.77	1.50 × 10^−3^	3.35 × 10^−2^
Phosphatidyl Glycerol (34:1)	3	747.522	3.17	↑	2.82	2.30 × 10^−3^	4.17 × 10^−2^
Triglyceride (33:0)	3	614.535	2.14	↓	0.12	1.00 × 10^−6^	9.60 × 10^−4^
Monoacylglycerol (24:0)	2	437.363	2.11	↓	0.11	2.00 × 10^−6^	9.66 × 10^−4^
Hexacosanoic acid	2	395.389	4.52	↓	0.17	1.30 × 10^−5^	2.27 × 10^−3^
Diacylglycerol (36:4)	3	675.520	5.00	↓	0.52	1.40 × 10^−5^	2.45 × 10^−3^
Monoacylglycerol (24:1)	2	439.378	2.09	↓	0.07	1.70 × 10^−5^	2.56 × 10^−3^
Monoacylglycerol (22:0)	2	413.363	1.98	↓	0.08	1.90 × 10^−5^	2.77 × 10^−3^
Sterol at C27H48O5	4	451.343	1.24	↓	0.09	4.00 × 10^−5^	4.22 × 10^−3^
Monoacylglycerol (22:4)	2	405.301	1.24	↓	0.06	4.90 × 10^−5^	4.93 × 10^−3^
Oxotestosterone (or steroid isomer)	3	320.218	0.79	↓	0.04	5.30 × 10^−5^	5.20 × 10^−3^
Triglyceride (28:0) Adduct	3	585.484	3.89	↓	0.04	8.90 × 10^−5^	7.13 × 10^−3^
Monoacylglycerol (22:2)	2	409.332	1.70	↓	0.11	1.59 × 10^−4^	1.01 × 10^−2^
Monoacylglycerol (24:4)	3	433.332	1.56	↓	0.10	2.27 × 10^−4^	1.22 × 10^−2^
Dihydrotestosterone (or Steroid isomer) sulphate	4	369.172	0.45	↓	0.13	4.85 × 10^−4^	1.80 × 10^−2^
Triglyceride (24:0)	3	493.355	2.76	↓	0.10	1.11 × 10^−3^	2.86 × 10^−2^
Capric acid	2	171.139	0.69	↓	0.22	1.32 × 10^−3^	3.13 × 10^−2^

## Supplementary Materials

The following are available online at https://www.mdpi.com/1422-0067/21/13/4753/s1, Table S1: Relative standard deviation (RSD%) of peak areas and retention times of a range of specific lipids representing major lipid families observed in plasma QC samples (*n* = 21), which were monitored during the analysis of the study samples.

## Abbreviations

PCOS	Polycystic Ovary Syndrome
EC	Endometrial Cancer
MVA	Multivariate Analysis
UVA	Univariate Analysis
PCA	Principal component analysis
OPLS-DA	Orthogonal projections to latent structures discriminant analysis
LC	Liquid chromatography
LC-MS	Liquid Chromatography-Mass Spectrometry
HRMS	High resolution mass spectrometry
