# Supplementary material for: Lipidomic Biomarkers in Polycystic Ovary Syndrome and Endometrial Cancer
Source: Int J Mol Sci. 2020 Jul 3;21(13):4753. doi: 10.3390/ijms21134753 (PMC7370092; doi:10.3390/ijms21134753)
Supplement: Supplementary file 1 [file ijms-21-04753-s001.pdf]

Supplementary Table

Table S1. Relative standard deviation (RSD%) of peak areas and retention times of a range of specific lipids representing major lipid families observed in plasma QC samples (n = 21), which were monitored during the analysis of the study samples.

Lipid Identity	*m/z*	%RSD Peak Area	%RSD Retention Time
Docosahexaenoic acid	327.232	7.6	0.62
α -or γ- Linolenic acid	277.217	7.3	0.31
LysoPC (20:4)	544.339	5.0	1.38
Arachidonic_acid	303.232	7.6	0.55
DiHETE	335.220	14.5	0.56
Palmitoleic acid	253.217	7.0	0.62
Docosapentaenoic acid	329.248	9.8	1.70
Linoleic acid	279.232	9.5	0.58
DHET	337.238	10.9	0.52
Palmitic acid	255.233	11.3	0.45
Oleic acid	281.248	11.6	0.42
Heptadecanoic acid	269.248	10.4	0.41
Stearic acid	283.263	9.8	0.30
Hexacosanoic_acid	395.389	7.3	0.58
Monoacylglycerol (22:4)	405.301	17.6	0.67
Triglyceride (24:0)	493.355	8.6	0.54
Triglyceride (28:0)	585.484	8.9	0.60
Triglyceride (52:3)	874.785	10.7	0.17
CE (18:2)	666.618	11.0	0.23
LysoPC (16:0)	496.340	7.8	1.53
LysoPC (18:0)	524.370	13.5	1.70
LysoPC (18:1)	522.355	8.4	1.70
LysoPE (18:0)	482.324	7.3	1.72
LysoPE (18:1)	480.309	6.6	1.76
LysoPE (22:6)	526.293	5.4	1.63
PC (36:6)	778.539	12.8	2.37
PG (34:1)	747.522	16.0	1.39
Ceramide: (d18:1/16:0)	596.526	9.3	0.74
Testosterone sulphate	367.158	14.1	1.71

LysoPC—lysophosphatidylcholine; LysoPE—lysophosphatidylethanolamine; HETE—hydroxyeicosatetraenoic acid; DHET—dihydroxyeicosatrienoic acid; PC—phosphatidylcholine; PG—phosphatidylglycerol; CE—cholesterol ester.
